# Cross-Validation of Next-Generation Sequencing Technologies for Diagnosis of Chromosomal Mosaicism and Segmental Aneuploidies in Preimplantation Embryos Model

**DOI:** 10.3390/life11040340

**Published:** 2021-04-12

**Authors:** Anil Biricik, Ettore Cotroneo, Maria Giulia Minasi, Pier Francesco Greco, Sara Bono, Matteo Surdo, Federica Lecciso, Mariateresa Sessa, Francesco Fiorentino, Francesca Spinella, Ermanno Greco

**Affiliations:** 1 Eurofins Genoma Group, Molecular Genetics Laboratories, Via Castel Giubileo 11, 00138 Rome, Italy; biricik@laboratoriogenoma.it (A.B.); cotroneo@laboratoriogenoma.it (E.C.); bono@laboratoriogenoma.it (S.B.); surdo@laboratoriogenoma.it (M.S.); lecciso@laboratoriogenoma.it (F.L.); sessa@laboratoriogenoma.it (M.S.); fiorentino@laboratoriogenoma.it (F.F.); 2 Villa Mafalda, Reproductive Medicine, 00199 Rome, Italy; mg.minasi@gmail.com (M.G.M.); p.greco753@gmail.com (P.F.G.); ergreco1@virgilio.it (E.G.); 3 Obstetrician and Genecology, UniCamillus International Medical University, 00131 Rome, Italy

**Keywords:** next generation sequencing, preimplantation genetic testing, chromosomal mosaicism, mosaic embryos, segmental aneuploidies

## Abstract

Detection of mosaic embryos is crucial to offer more possibilities of success to women undergoing in vitro fertilization (IVF) treatment. Next Generation Sequencing (NGS)-based preimplantation genetic testing are increasingly used for this purpose since their higher capability to detect chromosomal mosaicism in human embryos. In the recent years, new NGS systems were released, however their performance for chromosomal mosaicism are variable. We performed a cross-validation analysis of two different NGS platforms in order to assess the feasibility of these techniques and provide standard parameters for the detection of such aneuploidies. The study evaluated the performance of Miseq^TM^ Veriseq (Illumina, San Diego, CA, USA) and Ion Torrent Personal Genome Machine PGM^TM^ ReproSeq (Thermo Fisher, Waltham, MA, USA) for the detection of whole and segmental mosaic aneuploidies. Reconstructed samples with known percentage of mosaicism were analyzed with both platforms and sensitivity and specificity were determined. Both platforms had high level of specificity and sensitivity with a Limit Of Detection (LOD) at ≥30% of mosaicism and a showed a ≥5.0 Mb resolution for segmental abnormalities. Our findings demonstrated that NGS methodologies are capable of accurately detecting chromosomal mosaicism and segmental aneuploidies. The knowledge of LOD for each NGS platform has the potential to reduce false-negative and false-positive diagnoses when applied to detect chromosomal mosaicism in a clinical setting.

## 1. Introduction

With the recent advances in diagnostic technologies, comprehensive chromosome screening (CCS) has become a standard procedure in in vitro fertilization (IVF) treatment. The copy number of all chromosomes of a single blastomere, or of a multiple cells sample obtained from the trophectoderm can be determined in preimplantation genetic testing for aneuploidy (PGT-A) by means of CCS methods. The use of PGT-A is based on the assumption that transferring embryos identified with a normal genetic constitution (i.e., euploid) can improve clinical outcomes during IVF treatments [1,2]. Several studies have reported significantly higher implantation and reduced miscarriage rate after transfer of PGT-A screened embryos compared to embryo selection based only on morphological criteria [3,4,5]. Although, if the applied PGT-A technology is not adequately validated or the interpretation of the results is not accurate, then embryo screening can lead to reduced diagnostic accuracy of PGT-A. Consequently, this could potentially result in the expulsion of chromosomally normal embryos due to possible false positives, thus reducing the cumulative live birth rate [6].

In addition, a remarkable percentage of embryos diagnosed as euploid still cannot to progress to delivery. It has already been shown that implantation failure rates after euploid blastocysts transfer range from 25 to 50% [7,8]. Some of these failures could be explained with the presence of chromosomal mosaicism in these PGT-A screened embryo [9].

Chromosomal mosaicism may refer to embryos composed with normal and abnormal cell lines (e.g., euploid/aneuploid mosaic) or two or more different abnormal cell lines (e.g., aneuploidy-mosaic) [10,11]. In euploid/aneuploid mosaic embryos (hereafter simply referred to as ‘mosaic embryos’) mosaicism occurs by mitotic errors arising after fertilization of normal gametes. As first described by Greco et al. [12], despite their lower success rates than euploid embryos, the embryos with a PGT-A result suggesting mosaicism may have a potential for healthy pregnancies and births. These findings supported by others studies [13,14,15,16,17], so mosaic embryos have become highly relevant. Consequently, including the mosaic results in a PGT-A grading system as a separate category has been proposed [9] and endorsed by professional societies: PGDIS (Northbrook, IL, USA) and CoGEN (Brussels, Belgium) [18,19]. Recent studies demonstrated that the level and the types of mosaicism might influence developmental potential of mosaic embryos [13,14,15,16,17]. Thus, detailed interpretation of these mosaic profiles with PGT-A in a clinical trophectoderm analysis is therefore valuable and appropriate for embryo selection.

Nonetheless, there are still concerns regarding the diagnosis and interpretation of mosaicism in preimplantation embryos. False mosaic results could come from sub-optimal blastocyst biopsies and from technical back-ground noise due to amplification or sequencing artifacts and these may not be distinguishable from consistent mosaicism results [20]. Furthermore, most of the current analysis software leave the identification of mosaicism to the operator because of their analysis settings to classify only uniformly euploid or aneuploid samples, which may lead to subjectivity in diagnosing mosaicism with PGT-A. It has also been proposed that the cell cycle phase influencing readings by resembling mosaic segmental abnormalities could causes of artifactual mosaicism [21], although this effect appears to be minimized with contemporary, blastocyst-stage PGT-A methods based on trophectoderm (TE) cells biopsy [22].

Among various existing CCS-based PGT-A approaches, such as quantitative polymerase chain reaction (qPCR), comparative genomic hybridization (CGH), single-nucleotide polymorphism (SNP) arrays, and array-CGH [23], Next Generation Sequencing (NGS) has a comparatively high dynamic range and resolution, and is considered the most appropriate system for detecting aneuploidy and mosaicism [23,24,25,26].

Several NGS platforms were released, and at present the most popular NGS systems used for CCS are Miseq-Illumina (San Diego, CA, USA) [26] and Ion Torrent-Thermo Fisher (Waltham, MA, USA) [25]. These platforms are increasingly being used for the profiling of chromosomal aberrations within human embryos, including whole chromosome and segmental abnormalities, and monogenic diseases as well.

Due to its high dynamic range, NGS has the potential to accurately detect mosaic embryos and determine the percentage of aneuploidy cells in TE biopsy, providing an opportunity to improve detection of mosaicism within preimplantation embryos. However, each NGS methodology has a different capability to detect mosaicism levels due to different resolution capacity, so appropriate validation studies for chromosomal mosaicism are required.

The aim of this study was to validate and compare the capability of MiSeq-based (VeriSeq) and Ion PGM-based (Reproseq) NGS protocols to accurately detect segmental aneuploidy and chromosomal mosaicism in trophectoderm biopsies.

## 2. Results

### 2.1. Assessment of Chromosome Mosaicism by VeriSeq and ReproSeq NGS Methodologies in TE Biopsy Models

To assess the sensitivity and reproducibility of VeriSeq and ReproSeq-based NGS to detect chromosomal mosaicism we used artificially created models.

**Figure 1:**
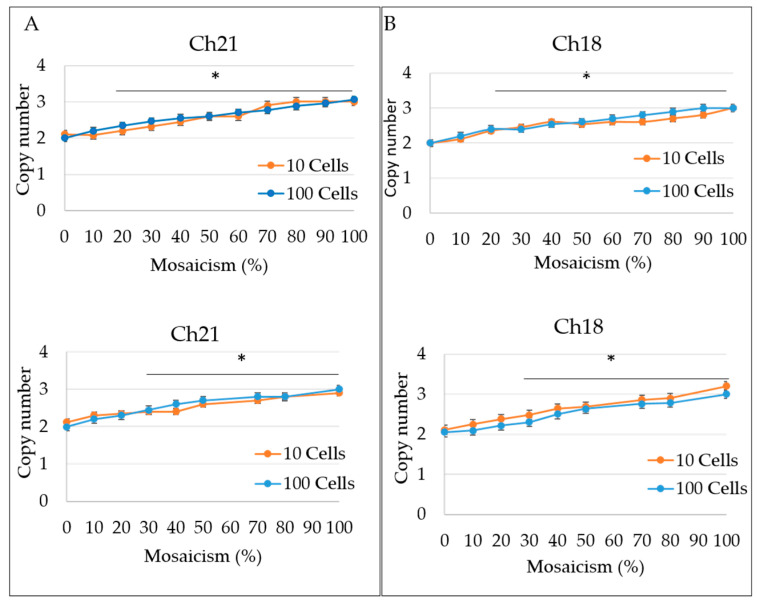
Reference curves obtained with reconstructed samples. The copy number of each dots (i.e., read count) for the mosaic chromosome was measured and the average value ± SD was correlated with the percentage of aneuploid cells present in each reconstructed samples. Top panel: result from MiSeq (VeriSeq); bottom panel results from Ion PGM (Reproseq). Reference curve obtained after the analysis of mosaic samples for chromosomes 21 (**A**) and 18 (**B**), obtained with 10 cell samples (orange line) and 100 cells samples (blue line). * *p* < 0.05 compared to euploid sample.

These were composed of a 100 cells set of reconstructed mosaic samples with different levels of mosaicism for representative chromosomes 18, 21, X, and Y which we first determine the reference curve for trisomic and monosomic mosaicism. For each level of mosaicism a copy number ± SD was defined (Figure 1 and Figure S1).

A second set of samples was generated to mimic different levels mosaicism in a blastocyst biopsy of 10 cells.

**Figure 2:**
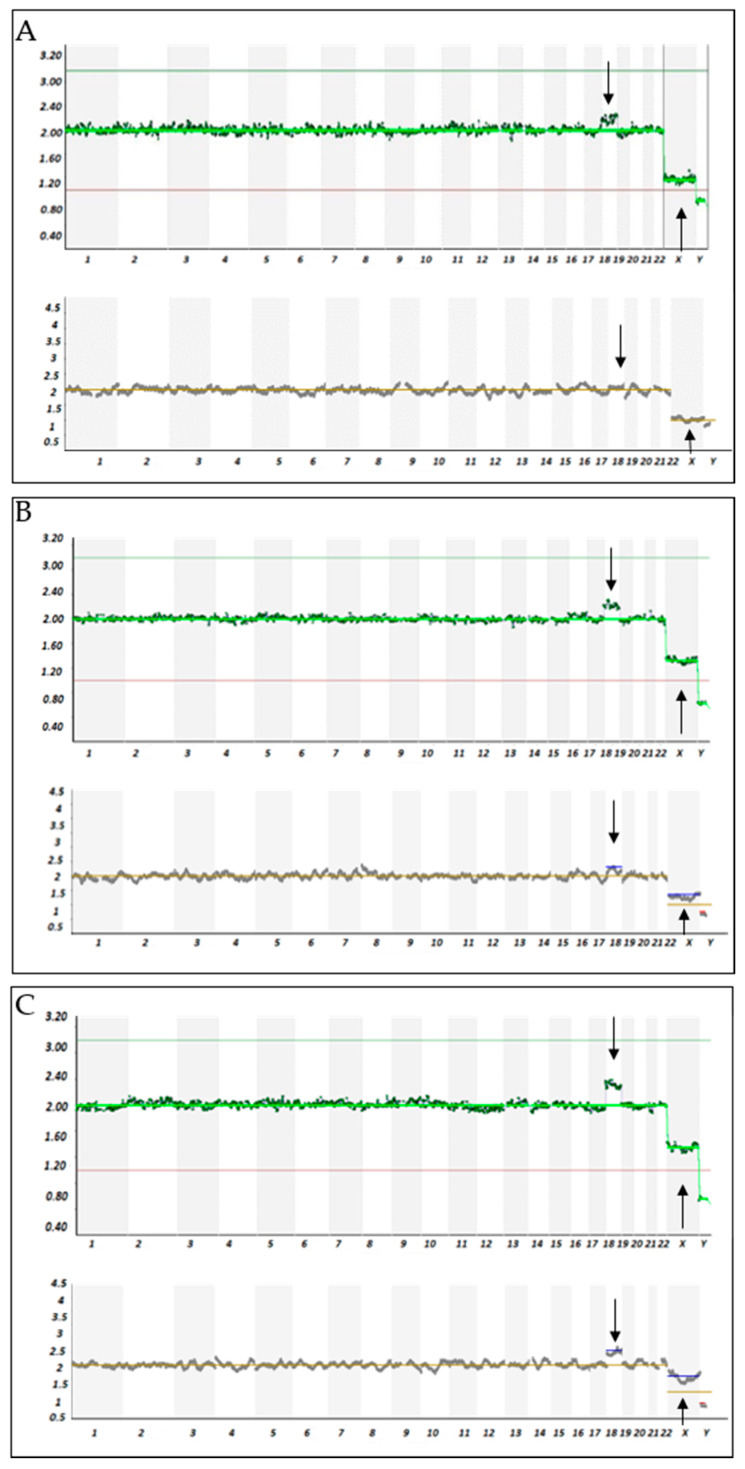
Examples of NGS results from mosaic reconstructed samples. Sample models with 20% (**A**), 30% (**B**), and 50% (**C**) mosaicism for representative chromosomes 18 and X. Top panel: result from MiSeq (VeriSeq); bottom panel: results from Ion PGM (Reproseq). NGS graphs indicates the copy number assignments (0, 1, 2, 3, or 4) on the *y*-axis and the chromosome number on the *x*-axis. Chromosomal mosaicism is seen as a shift of the dots (i.e., read count bins) between 2 and 3. Black arrows indicate chromosomes with chromosomal mosaicism.

With VeriSeq-based NGS a clear shift of copy number (CN) from the disomic level (CN = 2 ± 0.5) of the signal related to the chromosome involved in mosaicism was observed in samples ≥20% mosaicism. The CN of chromosome 21 (Chr21) and chromosome 18 (Chr18) increased from 2.2 to 2.8 concomitantly with the increases in aneuploidy cells from 20 to 80% in the reconstructed samples (Figure 1). A concomitant CN increase or decrease was observed for chromosome X and chromosome Y, respectively. Samples with 100% aneuploidy for trisomy 21 and 18, showed a 2.9 and 3 CNs, respectively (Figure 1). Likewise, for the 100% samples the ChX, and ChY CNs were 2.0 and 0.08, respectively (Figure S1). In addition, for all samples replicates, the other remaining autosomes showed the expected CNs of 2.0 (no mosaicism). Examples of NGS results are shown in Figure 2.

Manual calls were used to determine the CN of mosaic chromosome in each samples and the average values ± SD for Ch21, Ch18, and ChX mosaicism. Statistical analysis showed that the CNs of chromosomes with ≥20% mosaicism were significantly higher compared to those measured in non-mosaic samples. No significant statistical difference was observed for samples with 10% mosaicism demonstrating that VeriSeq is capable of detecting chromosomal mosaicism up to 20% level (Figure 1 and Figure 2, and Figure S1).

The same set of samples were analyzed with ReproSeq-based NGS. As shown in Figure 2 the CN of mosaic chromosomes increased with the increase in mosaicism percentage. Manual determination of CN for mosaic chromosomes in each samples showed no significant difference between chromosomes with 10 or 20% with those non-mosaic, while CN increased significantly (<0.05) in samples with chromosomal mosaicism ≥30% (Figure 2 and Figure S1). Statistical analysis demonstrated that ReproSeq-based NGS is capable of detecting chromosomal mosaicism up to 30% level (Figure 1 and Figure 2, and Figure S1).

### 2.2. Concordance Analysis

In total, 120 samples form 10 cells set of experiments were assessed for mosaicism detection for each NGS platform, 108 chromosomal mosaics (n = 54 for 46XY/47XX, +21 and n = 54 for 46XY/47XX, +18), 6 aneuploid (n = 3 for each set), and 6 euploid samples. All euploid, aneuploid, and 96 out of 108 of mosaic samples were correctly classified with VeriSeq-based NGS. The 12 false negative results obtained with NGS came from samples with 10% mosaicism. There were no false positive diagnoses for euploid samples.

The VeriSeq-based NGS results were then compared for consistency at chromosomal levels. In total 2880 chromosomes of which 330 aneuploid (n = 162 for each set of experiment): the number of true positive chromosome, i.e., Ch18/Ch21, ChX, ChY, for each mixture level plus 6 full aneuploidy chromosomes, i.e., Ch18 and Ch21were assessed. Of the 330 aneuploid, 294 samples resulted with a copy number alteration. VeriSeq-based NGS specificity for aneuploidy call (consistency of chromosome copy number assignment) was 100.00% (confidence interval-CI 95%; 99.86 to 100.00%) with a sensitivity of 90.16% (CI: 86.64 to 93.02%). The 36 false negative results came from samples with a mosaicism of 10% (Table 1).

With ReproSeq-based NGS, 258 out of 330 resulted with a copy number imbalance. The ReproSeq-based NGS specificity for aneuploidy call (consistency of chromosome CN assignment) was 100% (95% CI 99.86–100) with a sensitivity of 82.09% (258 out of 330, 95% CI: 77.98–85.71). The 72 false negative results came from samples with a mosaicism of 10% (n = 36) and 20% (n = 36) (Table 1).

For aneuploid sample calling (24-chromosome diagnosis consistency), sensitivity and specificity were also calculated and are reported in Table 1.

These results demonstrated that both NGS platforms are capable to detect mosaicism but showed different sensitivity.

### 2.3. Assessment of Segmental Aneuploidies by VeriSeq and ReproSeq-NGS Methodologies in TE Biopsy Models

**Figure 3:**
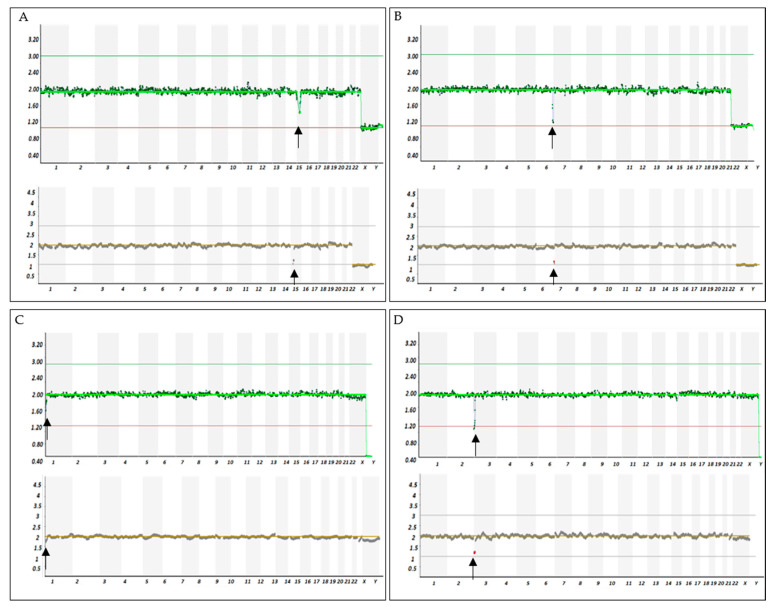
Examples of NGS results from samples with segmental abnormality. On the y-axis is indicated the copy number (0, 1, 2, 3, or 4) and on the x-axis the chromosome number. Graphic representation of copy number changes observed in the cell line samples with a microdeletion of 5.04 Mb (Ch15) (**A**), 7.9 Mb (Ch6) (**B**), 4.5 Mb (Ch1) (**C**) and 10.0 Mb (Ch3) (**D**). Top panel: result from MiSeq (VeriSeq); bottom panel: results from Ion PGM (Reproseq). Chromosomal structural abnormality (deletion) is seen as a shift of the dots (i.e., read count bins) between 1 and 2. Black arrows indicate chromosomal deletion.

A total of 16 samples (duplicates of 8 cell lines with different structural abnormalities) were analyzed with VeriSeq and ReproSeq (Table S2). After manual assessment VeriSeq detected 16/16 segmental aneuploidies. No additional whole or segmental imbalances were identified. With ReproSeq, segmental chromosomal calls were assigned by the software for 14 out of 16 samples. No additional whole or segmental imbalances were identified. One sample with segmental aneuploidy of 4.5 Mb was not detected with ReproSeq. Examples of VeriSeq and ReproSeq are shown in Figure 3.

The results obtaining from samples with segmental aneuploidy cell lines demonstrated that ReproSeq could identify a segmental imbalance with 5.0 Mb in size, while VeriSeq could identify microdeletion as small as 4.5 Mb.

**Figure 4:**
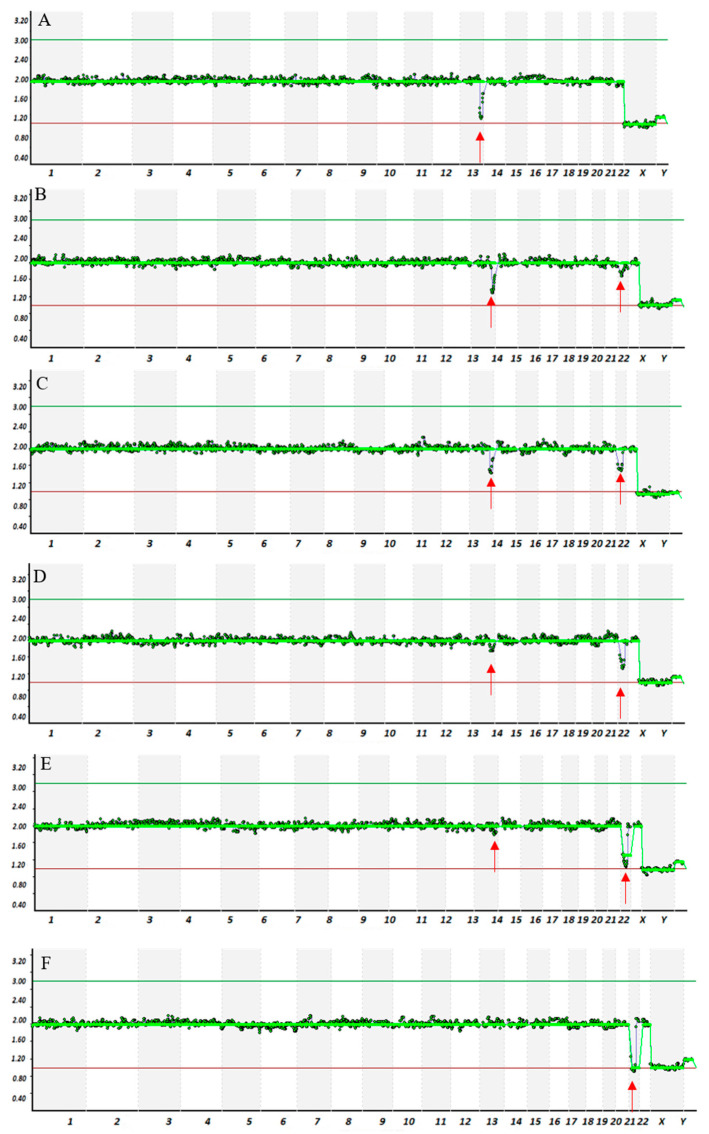
Examples of VeriSeq-NGS results from samples with mosaic structural abnormality. On the y-axis is indicated the copy number (0, 1, 2, 3, or 4) and on the x-axis the chromosome number. Graphic representation of copy number changes observed in the cell line samples with a microdeletion of 12 Mb (Ch13), and 17 Mb (Ch21). (**A**) sample model with 100% of the cells with structural aneuploidy for the 12 Mb (Ch13) and 0% of cells with 17 Mb (Ch21); (**B**) sample with 80% of cells with 12 Mb (Ch13) and 20% with 17 Mb (Ch21); (**C**) sample with 60% of cells with 12 Mb (Ch13) and 40% with 17 Mb (Ch21); (**D**) sample with 40% of cells with 12 Mb (Ch13) and 60% with 17 Mb (Ch21); (**E**) sample with 20% of cells with 12 Mb (Ch13) and 80% with 17 Mb (Ch21); (**F**) sample with 0% of cells with 12 Mb (Ch13) and 100% with 17 Mb (Ch21). Red arrows indicate chromosomal deletion.

Assessment of mosaic segmental aneuploidies by VeriSeq-based NGS confirmed the capability of this methodology to detected mosaicism also for segmental aneuploidies. In these samples, segmental errors were observed as the percentage of aneuploidy cells was 20%. As shown in Figure 4, the copy number for 17 Mb (Chr21) deletion was 2.0 copy in sample model with 100% of euploidy cells and decreased gradually toward the 1.0 copy in higher aneuploidy cells models. A parallel increase in the copy number for 12 Mb (Chr13) deletion was observed from sample with 100% aneuploidy to sample with 20% mosaicism (Figure 4).

## 3. Discussion

Our findings demonstrated that NGS methodologies are capable of accurately detecting chromosomal mosaicism and segmental aneuploidies; however, the limit of detection (LOD) for each NGS platform differs. The knowledge of LOD for each NGS platform has the potential to reduce false-negative and false-positive diagnoses when applied to detect chromosomal mosaicism.

Our findings support the use of the two major platforms commercially available for NGS-based PGT-A for the detection of mosaicism and segmental aneuploidies in trophectoderm biopsies and demonstrate that VeriSeq NGS has a slightly higher resolution for segmental aneuploidies and a higher level of accuracy at a 20% level of mosaicism compared to ReproSeq.

Until recently, mosaicism has been difficult to detect. Indeed, mosaicism ranging from 40 to 60% can be detected with a high degree of confidence with the use of methods such as array-CGH and qPCR, but they are relatively insensitive for the detection of low-level mosaicism [20,27,28]. This could have led to viable embryos being inappropriately discarded or the inadvertent transfer to the uterus of aneuploid embryos [20,29].

NGS platform has the potential to overcome this limitation, providing an opportunity to improve IVF clinical outcomes.

However, NGS and data analysis pipelines used to measure chromosome copy number variation may in some embryos incorrectly indicate mosaicism because of various technical effects, causing false positive results. Technical errors or artifacts are expected to be introduced by the whole genome amplification (WGA) technique used to amplify embryonic DNA [20,30] or could result from the method of biopsy. Biopsy methodology and the number of retrieved cells (i.e., less than 5) may affect amplification profiles (noise) and mosaic detection levels. In addition, cell damage or partial destruction and loss of cellular DNA obtained from biopsy may alter chromosome profiles. Analysis of the results could also induce artifacts; the algorithms used for normalizing the chromosome mapping bins can potentially alter profiles, especially if bin counts used to normalize the profiles are not available or few. In addition, poor DNA quality could lead to under or over representation of chromosomes (whole chromosome mosaicism) or sub-chromosomal regions (segmental mosaicism) [30,31]. Recently preimplantation genetic testing consortium released specific guideline to help laboratories to reduce the risk of misdiagnosis when facing with mosaic embryos [32]. It has been suggested that 5–10 cells should be biopsied to give subsequent robust and balanced amplification of DNA and cell damage should be minimized to reduce amplification bias. This because more cells (DNA) in the test specimens, more accurate prediction for the copy numbers could be achieved. PGDIS recommendation also suggests that only NGS platform that can reproducibly measure copy number should be used for reporting of mosaic levels in the biopsy sample [18]. In the recent years, new NGS platforms were released and their performance for segmental aneuploidies and low level of chromosomal mosaicism, as well as intrinsic baseline noise level, are variable. For these reasons detection and quantification limits of mosaic level should be defined for any platform.

In this study, we performed validation study for chromosomal and segmental aneuploidy detection for the two NGS platforms, addressing the capability of each to detect low percentage of mosaicism and segmental aneuploidies. To this end, we used a set of reconstructed mosaic samples and cells with different segmental aneuploidy. In reconstructed mosaic experiments, different proportions of aneuploid cells (from 0 to 100%) could be discriminated from one another in all cases, indicating that the different NGS platforms were not only capable of detecting mosaicism, but also have the potential to quantify the proportion of aneuploid cells within a sample with a known number of cells. In addition, we provide the LOD for each NGS methodology. Specifically, we found a LOD of ≥20% for VeriSeq and ≥30% for ReproSeq platform. Regarding the resolution for segmental abnormalities, VeriSeq and ReproSeq identify microdeletion as little as 4.5 and 5.0 Mb in size, respectively. However, it is important to note that the study has been done with the current versions of both NGS platforms and eventual upgrades on algorithms of both systems may improve the sensitivity and specificity.

When assessing the capability of NGS platforms to detect mosaic segmental aneuploidy we found that VeriSeq is able to accurately detect small segments (12–17 Mb) in samples composed with >20% of aneuploidy cells.

The performance of VeriSeq-based NGS to detect whole and partial chromosomal mosaicism has been recently described by Goodrich et al. [33,34]. In concordance with our results, the authors reported a LOD of 17% mosaicism when they applied a custom VeriSeq analysis criteria, as defined by other authors [35]. However, applying these criteria the false positive rate increased from 0% for samples with >50%, to a 67% for those <50% mosaicism. In contrast with Goodrich results, we did not detect false positive results in sample ≥20% mosaicism neither for whole or segmental mosaic aneuploidies. Although, we are unable to provide an explanation for such discrepancy, we certainly recognized that detection of low-grade mosaicism within an embryo may be subject to some degree of sampling error. However, proper validation of NGS system reduce the potential risk of bias possibly deriving from whole genome amplification (WGA) artifacts. That is why it is essential to perform cell lines mixing experiments simulating chromosomal mosaicism. Specifically, in our experiments we ran a wide number of fully euploid (only euploid cells, 2 copies of each chromosome) samples, in order to define the standard deviation from the euploid baseline value (2 copies value). Any chromosome copy number value that falls outside this range (euploidy range) was scored as mosaic. The mixing experiment with euploid and aneuploid cell lines at different percentage of aneuploid vs. euploid cells was used to define the LOD of the NGS system, i.e., the lower aneuploidy percentage detectable by the specific NGS system, involving a copy number value that will fall outside the euploidy range. Ideally, every methodology intended to detect the presence of mosaicism with small numbers of cells should first be validated on a large dataset of single cells and with mosaic reconstructed samples (positive controls).

One of the limitation of our study is that the number of 10 cells per samples used in our experimental models could be higher than what most of clinical embryologists will obtain during TE biopsy. Typically, most of them may take 5–8 cells from a Day5 or Day6 embryos, thus it could be more challenging to detect changes that represent less than 20–30% of the biopsy. This should be considered when interpreting the result in a clinical setting. A second limitation of our study is that we analyzed aneuploidy only for chromosomes 13, 21, and X, and our results may not completely be applicable to other chromosomes. It should be minded that resolution may vary from one chromosomal region to another due to variations in the NGS read coverage. For this reason, validation experiment especially to other small chromosomes, such as chromosome 19, 20, 22, and Y should be performed to define the exact condition to detect mosaicism for all chromosomes.

Sensitivity and specificity that we obtained specifically apply for the described NGS platforms (hardware and protocol for WGA or library preparation for NGS) and software or bioinformatics paradigm used to analyze the data and these cannot be exchanged among platforms.

These data provide much needed evidence-based guidelines for appropriate validation study for accurate detection of mosaic embryos in the clinic. Parameters obtained from this study were used for diagnosing more than 2000 mosaic embryos obtained in our clinic from May 2019 to December 2020 [36]. NGS-based PGT-A detected different types and level of mosaic embryos, including embryos with whole-chromosome mosaicism, segmental (or partial), complex or a combination of such aneuploidies and embryos with a percentage of aneuploidy cells ranging between 20% and 70%. Clinical outcomes of 300 of these mosaic showed a significant reduction in ongoing clinical pregnancy and baby’s birth rate compared to full euploid embryos. Whole chromosome mosaic embryos with mosaicism below 50% had significantly more favorable outcomes than the ≥50% group. In addition, mosaic embryos with segmental abnormalities or single aneuploidy showed higher ongoing pregnancy rate compared to mosaic with complex aneuploidies affecting three or more chromosomes. Of note, for specific type of chromosomal mosaicism (i.e., segmental abnormalities) there was no difference in clinical outcome between low (20%) to moderate mosaicism (30%), suggesting that starting point for reporting mosaicism could be 30%. However, a statistically significant difference in clinical outcomes was observed in low vs. moderate whole-chromosome mosaicism [37]. Such evidences emphasize the importance to detect and report also low level of mosaicism during PGT analysis in a clinical TE biopsy for an appropriate genetic counselling and valuable selection of embryo [9,37,38,39].

Our results emphasize the importance of vigorous preclinical evaluation of NGS-based PGT-A methodologies with specific criteria for mosaicism detection prior to clinical implementation. This kind of validation should be performed for each NGS platforms and laboratory as internal variation may occur for a variety of factors. The lack of such vigorous preclinical evaluation will certainly impair a correct PGT-A analysis especially when applied for the detection of mosaicism within preimplantation embryos.

## 4. Materials and Methods

### 4.1. Experimental Design

The study was designed into three steps.

In the first step we created the reference curves for different level of mosaicism. To this end we used the set of 100 cells with different proportion of euploid and aneuploid cell mixture ranging from 10% to 100% (Table S1) and analysis with both VeriSeq and ReproSeq based NGS platforms.

The second step was the validation of the NGS methodologies for chromosomal mosaicism and the definition of the mosaicism detection level or limit of detection (i.e., the minimum ratio of aneuploid to euploid cells which is needed to detect a copy number variation (CNV)).

The third step was to assess the resolution of both NGS platforms for segmental aneuploidy detection. The eight different cell lines (Coriell Cell Repository, Camden, NJ, USA) with specific structural abnormalities (changing from 4.5 to 17 Mb) were used for segmental aneuploidy assessment (Table S2). The aim was to define the resolution limit of the methodologies.

In addition, we provided a model with different levels of segmental mosaicism which can be expected to observe in a typical trophectoderm biopsy and assessed the ability of NGS to detect sub-chromosomal imbalances for segmental aneuploidy in a mosaic example. For this aim a mixture of two different cell lines with 12 and 17 Mb microdeletion were used to mimic different levels of mosaic segmental aneuploidies (20, 40, 60, and 80% mosaicism). Duplicates of each cell mixture were prepared and analyzed with VeriSeq-based NGS protocol.

### 4.2. Reconstructed Mosaicism Experimental Model

The isolation of cell samples (46, XY; 47, XX, +18 and 47, XX, +21) has been done by using a flow sorter (FACS Aria II SE, (BD Biosciences, San Jose, CA, USA) and the samples were mixed in the following proportions: 10 or 100 aneuploid cells from 0 to 100% euploid cells; 10:0; 9:1; 8:2; 7:3; 6:4; 5:5; 4:6; 3:7; 2:8;1:9, and 0:10 (Table S1). Identically proportioned duplicated cell lines were lysed and processed for VeriSeq and ReproSeq-based NGS analysis according to manufacturer’s instructions.

For segmental aneuploidy mosaicism reconstruction, two cell lines (Coriell Cell Repository, Camden, NJ, USA) containing known segmental deletions, GM08331 [46, XY, del (13) (pter->q31:q34->qter). arr [hg19] 13q32.1q33.3 (98,158,969 − 110,263,569) × 1, 21q21.3 (27,316,123 − 29,519,188) × 1] and GM06918 [46, XY, del (21) (q11.2q22). ish del (21) (wcp21+). arr 6q26 (162,784,828 − 162,990,795) × 1, 21q11.2q22.11 (15,275,679 − 32,592,618) × 1], were used in the following proportions: 10 aneuploid cells to 0 euploid cells; 8:2; 6:4; 4:6; 2:8, and 0:10 (Table S1). Duplicates of each mix were obtained and analyzed with VeriSeq-based NGS protocol. The karyotype of each cell line was provided by the supplier.

### 4.3. VeriSeq-NGS Protocol (Illumina)

#### 4.3.1. Whole Genome Amplification

For WGA, genomic DNA first extracted from the cells by lysis and then fragmented randomly and amplified using the SurePlex DNA Amplification System (Illumina Inc., San Diego, CA, USA), according to the manufacturer’s protocol. This proprietary single tube reaction technology is based on genomic DNA random fragmentation and following PCR amplification utilizing flanking universal priming sites, as previously described [26].

Briefly, cells collected in 2.5 μL of 1× PBS were lysed using 2.5 μL of SurePlex cell extraction buffer and 5 μL of the SurePlex Extraction cocktail master mix with incubation at 75 °C for 10 min, the sample were then incubated at 95 °C for 4 min. The random fragmentation of genomic DNA was carried out by adding 5 μL of SurePlex Pre-amplification mixture to the lysed cell samples or to genomic DNA controls and incubating the mixture as follow: one cycle of 95 °C for 2 min, followed by 12 cycles of 95 °C for 15 s, 15 °C for 50 s, 25 °C for 40 s, 35 °C for 30 s, 65 °C for 40 s, and 75 °C for 40 s, followed by a hold at 4 °C. After this, 60 μL of freshly prepared Sureplex Amplification mixture was added to 15 μL of synthesis product in each reaction tube. Resulting mixtures were amplified according to the following thermal cycler program: one cycle of 95 °C for 2 min, followed by 14 cycles of 95 °C for 15 s, 65 °C for 1 min and 75 °C for 1 min, followed by a hold at 4 °C. To assess the success of the amplification, 5 μL of each amplified sample plus 5 μL gel loading buffer were examined by electrophoresis on a 1.5% agarose 1× Tris-Borate-EDTA(TBE) gel. DNA amplification products were then quantified using the Qubit^®^ dsDNA HS Assay Kit (Life Technologies Corporation, Grand Island, NY, USA).

#### 4.3.2. NGS Analysis

Libraries were prepared using the VeriSeq PGS workflow (Illumina, Inc., San Diego, CA, USA). DNA ‘indexing’ was performed using the Veriseq Index Kit-PGS (Illumina, Inc., San Diego, CA, USA). During the library preparation step, the input DNA was tagged and fragmented by the NexteraTM XT transposome. The Nextera transposome simultaneously fragments the input dsDNA and adds adapter sequences to the ends, allowing amplification by PCR in subsequent steps. A limited-cycle PCR reaction uses these adapter sequences to amplify the insert DNA. The PCR reaction also adds index sequences on both ends of the DNA, thus enabling dual-indexed sequencing. In total, 1 ng of quantified dsDNA template at 0.2 ng/µL was added to 5 µL of Amplicon Tagmentation Mixture and 10 µL of Tagmentation DNA Buffer. The tagmented DNA was amplified via a limited-cycle PCR. PCR product clean-up used AM Pure XP beads (BeckamCoulter, Brea, CA, USA) to purify the library DNA. Purified libraries were eluted with 50 µL of the Nextera XT Resuspension Buffer. Each indexed library was normalized by beads and then multiplexed in 24-plex library pools.

Single-end, dual index 36 base pair reads (1 × 36 double index) sequencing was performed using the Illumina v3 chemistry workflow on a MiSeq sequencer with the MiSeq Reagent Kit v3-PGS (Illumina, Inc., San Diego, CA, USA), which contains the ready to load on-board clustering and sequence by synthesis (SBS) chemistry reagents. A sample sheet, used by both the MiSeq system and Bluefuse software, was generated using BlueFuse Workflow Manager. Reads were demultiplexed and aligned to the human genome hg19 by the on-instrument MiSeq Control Software (MCS v2.5, Illumina, Inc, San Diego, CA, USA). Binary Alignment Map (BAM) files from the MiSeq system are imported directly into the BlueFuse Multi (4.3) analysis software (Illumina, Inc., San Diego, CA, USA) using the prepared sample sheet. The software processes and displays the data to provide genomic profiles of each sample in a run. The samples acceptance criteria was a number of total reads >700,000 with a number of reads passing filter >500,000, and overall noise (DLR) ≤0.2.

The count data in each bin was normalized using GC content and in silico reference data in order to remove bias, and CN were determined using of a combination of a Gaussian probability function (PDF; with copy number states 0–4 and a standard deviation of 0.33) and thresholding [26]. The CN state with the highest probability for a chromosome was used unless the distance to the next most probable copy number was >0.011. In that case, the median value of the most likely copy number states of all bins of a chromosome was used, set to a gain when >2.5 and to a loss when <1.5.

### 4.4. Reproseq NGS Protocol (Thermo Fisher)

#### 4.4.1. Whole Genome Amplification and Library Preparation

Ion SingleSeq Kit (Ion ReproSeq PGS Kit) was used for WGA and barcoding of library according to the manufacturer’s instructions. Cell samples were lysed in 2.5 μL of cell extraction buffer and 5 μL of the extraction master mix with incubation at 75 °C for 10 min followed by incubation at 95 °C for 4 min. The random fragmentation of genomic DNA was carried out by adding 5 μL of pre-amplification master mix to the lysed cell samples or to genomic DNA controls and incubating the mixture according to the following protocol: one cycle of 95 °C for 2 min, followed by 12 cycles of 95 °C for 15 s, 15 °C for 50 s, 25 °C for 40 s, 35 °C for 30 s, 65 °C for 40 s, and 75 °C for 40 s, followed by a hold at 4 °C. After the adding 30 μL of freshly prepared amplification master mix on each sample, barcoding has been done by adding 5 μL of SingleSeq Barcode Adapters (SingleSeq Barcode Set 1) on appropriate samples. Library amplification has been done according to the following PCR program: one cycle of 95 °C for 3 min, followed by 4 cycles of 95 °C for 20 s, 50 °C for 25 s, and 72 °C for 40 s, followed by 12 cycles of 95 °C for 20 s, 72 °C for 55 s, and final hold at 4 °C. Unamplified barcoded fragments were size selected using an E-Gel^®^ SizeSelect™ 2% agarose system running until the 300-bp band of the 50-bp ladder (Invitrogen) reached the marked reference lines on the E-Gel^®^ cassette. The final concentration of the libraries was normalized to 100 pM using the Ion Library Equalizer Kit (Life Technologies, Carlsbad, CA, USA). The normalized barcoded libraries were pooled in a 24-plex manner and purified by Agencourt AMPure XP Beads (Beckman Coulter, Brea, CA, USA). The library pool then quantified by Qubit^®^ dsDNA HS Assay Kit (Life Technologies, Carlsbad, CA, USA) and diluted to 1 nM by nuclease-free water.

#### 4.4.2. Template Preparation and Enrichment

Sequencing templates on IonSpare PraticlesTM have been prepared by isothermal amplification using Ion PGM Template IA 500 Kit (Ion ReproSeq). Then, 10 pM library pool firstly heated at 70 °C for 2 min and hold at 4 °C until IA reaction. The templating solution has been prepared according to the manufacturer’s instructions starting from 10 µL of 10 pM normalized library pools and was used with Ion PGM IA Pellets for a clonal amplification of libraries on Ion Sphare Particles. Isothermal amplification was done on a pre-heated heat block for 25 min at 40 °C and reaction was terminated by Ion PGM Template IA Stop Solution. Template-positive Ion Sphere^TM^ Particles (ISPs) were then recovered by Ion PGM Template IA Recovery and Ion PGM Template IA Wash solutions. The enrichment of template-positive ISPs was performed on the Ion OneTouch™ ES system according to the manufacturer’s protocol.

#### 4.4.3. NGS Analysis

Templated spheres were loaded on an Ion 318™ chip, and sequencing was performed on an Ion PGM™ running Torrent Suite™ Software 5.0.5 (Thermo Fisher, Waltham, MA, USA) using the Ion PGM Hi-Q Sequencing Kit.

Data were automatically uploaded to Ion Reporter™ Software 5.10 (Thermo Fisher, Waltham, MA, USA) and analyzed using a Low-pass whole-genome aneuploidy workflow, an algorithm that allows copy cumber analysis from a test sample, based on the ratio between the percentage of reads derived from a given chromosome test and a reference value for the same chromosome. As reference value, a custom baseline generated from 11 normal samples was used according to the Ion Reporter™ user manual. A custom baseline was also adopted to reduce the amplification bias and optimize the detection of CNV and consisted of a reference value for each chromosome calculated by averaging the percentage of mapped reads in a series of euploid samples. The CN value with the highest probability for a chromosome was assigned a confidence value of 10. The CNV coverage data were also visualized in the Integrative Genomics Viewer genome browser, launched directly from Ion reporter, which graphically shows the difference in test and control coverages in a ploidy-centric Y-axis. Chromosomal gains were associated with a CN >3 and losses with a copy number <1 [25].

#### 4.4.4. Concordance Analysis

CN calls automatically generated by Bluefuse Multi™ 4.3 (Illumina. Inc, San Diego, CA, USA) or by Ion Reporter™ 5.10 (Thermo Fisher, Waltham, MA, USA) software (company, city, state (for USA), country), then were assessed manually and compared for sample ploidy status, sample karyotype, and chromosome ploidy status.

The results were compared to the karyotype of cells and the concordance was calculated with the use of the classifications true positive (TP; gain or loss call detected), true negative (TN; euploidy status confirmed), false negative (FN; gain or loss call missed), and false positive (FP; additional gain or loss called).

#### 4.4.5. Evaluation of Sensitivity and Specificity

After the predictions of chromosomal mosaicism and segmental aneuploidy were made, reconstructed samples were evaluated for consistency with expected results. Sensitivity was defined as the percentage of samples which were predicted as abnormal for the correct chromosome (n = 114; 108 chromosomal mosaics samples plus 6 full aneuploid samples) or segmental aneuploidy (n = 8). Specificity was defined as the percentage of samples where euploidy was predicted for all the chromosomes and segments expected to be normal or disomic (n = 6). The sensitivity, and specificity, of the test were calculated as follows:

Specificity: number of true negatives/(number of true negatives + number of false positives);

Sensitivity: number of true positives/(number of true positives + number of false negatives).

Sensitivity defines the probability that the aneuploidy call will be positive when aneuploidy is present (true positive rate). Specificity defines the probability that the aneuploidy call will be negative when aneuploidy is not present (true negative rate).

A sample was classified as euploid when all chromosomes showed a CN value within the normal ploidy range. A sample was classified as aneuploid or mosaic when the CV was between diploid and triploid or monosomic values for one or more chromosome. A sample was classified as diploid and aneuploid mosaic when chromosomal mosaicism and no aneuploidy on other chromosomes was detected in the same sample.

### 4.5. Statistical Analysis

Results were reported as average ± standard deviation (SD) from at least three replicated experiments for each group of interest. Chromosome copy number values for each percentage of mosaicism in different sets of experiments were compared using *t*-test with corresponding *p* values for each comparison made. *p* values were determined to be significant at *p* < 0.05 using PRISM software (GraphPad Software, Inc., San Diego, CA, USA).

## Figures and Tables

**Table 1:** Concordance analysis.

Concordance Analysis	VeriSeq No. (95% CI)	ReproSeq No. (95% CI)
Chromosome calling comparison	2888	2888
Euploid chromosomes (true negatives)	2550	2550
Aneuploid chromosomes (true positives)	330	330
Missed chromosome calls (false negatives)	36	72
Extra chromosome calls (false positives)	0	0
Aneuploidy call performance		
Sensitivity	90.16% (86.64–93.02%)	82.09% (77.98–85.71%)
Specificity	100% (99.86–100%)	100% (99.86–100%)
Whole-embryo Aneuploidy/Euploidy status comparison		
Euploid sample (true negatives)	6	6
Aneuploid embryo (true positives)	114	114
Missed aneuploid embryo calls (false negatives)	12	24
Extra aneuploid embryo calls (false positives)	0	0
Aneuploid embryo call performance		
Sensitivity	94.48% (83.95–94.98%)	82.61% (75.24–88.53%)
Specificity	100% (54.07–100%)	100% (54.07–100%)

## Data Availability

Not applicable.

## Supplementary Materials

The following are available online at https://www.mdpi.com/article/10.3390/life11040340/s1, Figure S1: Reference curves obtained with reconstructed samples. Table S1: Reconstructed samples for whole and segmental chromosome mosaicism. Table S2: Characteristic of segmental aneuploidies and NGS results.
