# Supplementary material for: Cross-Validation of Next-Generation Sequencing Technologies for Diagnosis of Chromosomal Mosaicism and Segmental Aneuploidies in Preimplantation Embryos Model
Source: Life (Basel). 2021 Apr 12;11(4):340. doi: 10.3390/life11040340 (PMC8069536; doi:10.3390/life11040340)
Supplement: Supplementary file 1 [file life-11-00340-s001.pdf]

Supplementary Materials

Cross-Validation of Next-Generation Sequencing Technologies for Diagnosis of Chromosomal Mosaicism and Segmental Aneuploidies in Preimplantation Embryos Model

Table S1. Reconstructed samples for whole and segmental chromosome mosaicism.

Mosaicism (%)	0	10	20	30	40	50	60	70	80	90	100	Total
**No. of samples (46,XY/47,XX+21)**	3[a]	6[a]	6[a]	6[a]	6[a]	6[a]	6[a]	6[a]	6[a]	6[a]	3[a]	60
	-	3[b]	3[b]	3[b]	3[b]	3[b]	3[b]	3[b]	3[b]	3[b]	3[b]	30
**No. of samples (46,XY/47,XX+18)**	3[a]	6[a]	6[a]	6[a]	6[a]	6[a]	6[a]	6[a]	6[a]	6[a]	3[a]	60
	-	3[b]	3[b]	3[b]	3[b]	3[b]	3[b]	3[b]	3[b]	3[b]	3[b]	30
**No. of samples [46,XY, del 13 (12Mb)/46,XY,del21 (17Mb)]**	2		2		2		2		2		2	12

a 10 cells set, b 100cells set

Table S2. Characteristic of segmental aneuploidies and NGS results.

	Cell Lines	Chromosomal Rearrangement	Size	VerySeq *	Reproseq *
1	GM22991	46,XX.ish del(1)(p36.32)(CEB108/T7-,SKI-,D1S3739+).arr 1p36.32(742429 - 5215341)x1	4.5 Mb	2/2	0/2
2	GM11382	46,XY,del(15)(q11.2q13).ish del(15)(q11.2q13)(D15S11-,GABRB3-).arr 15q11.2q13.1(21192942 – 26234399)x1	5.04 Mb	2/2	2/2
3	GM22624	46,XX,del(11)(p12p11.2).arr 11p12p11.2(40433344-46031324)x1	5.6 Mb	2/2	2/2
4	GM111516	46,XX.ish del(15)(q11.2q13)(D15Z1+,D15S10-,PML+).arr 15q11.2q13.1(19803357 – 26872582)x1	7.07 Mb	2/2	2/2
5	GM21698	46,XY,del(6)(q26).ish del(6)(q26)(wcp6+,D62522-).arr 6q26q27(162860228 – 170761408)x1	7.9 Mb	2/2	2/2
6	GM10985	46,XX,del(3)(p25).arr 3p26.3p25.3(35333-10305377)x1	10 Mb	2/2	2/2
7	GM08331	46,XY,del(13)(pter->q31::q34->qter).arr[hg19] 13q32.1q33.3(98158969-110263569)x1,21q21.3(27316123-29519188)x1	12 Mb	2/2	2/2
8	GM06918	46,XY,del(21)(q11.2q22).ish del(21)(wcp21+).arr 6q26(162784828-162990795)x1,21q11.2q22.11(15275679-32592618)x1	17 Mb	2/2	2/2
Tot				16/16	14/16

*number of detect/number of investigated samples

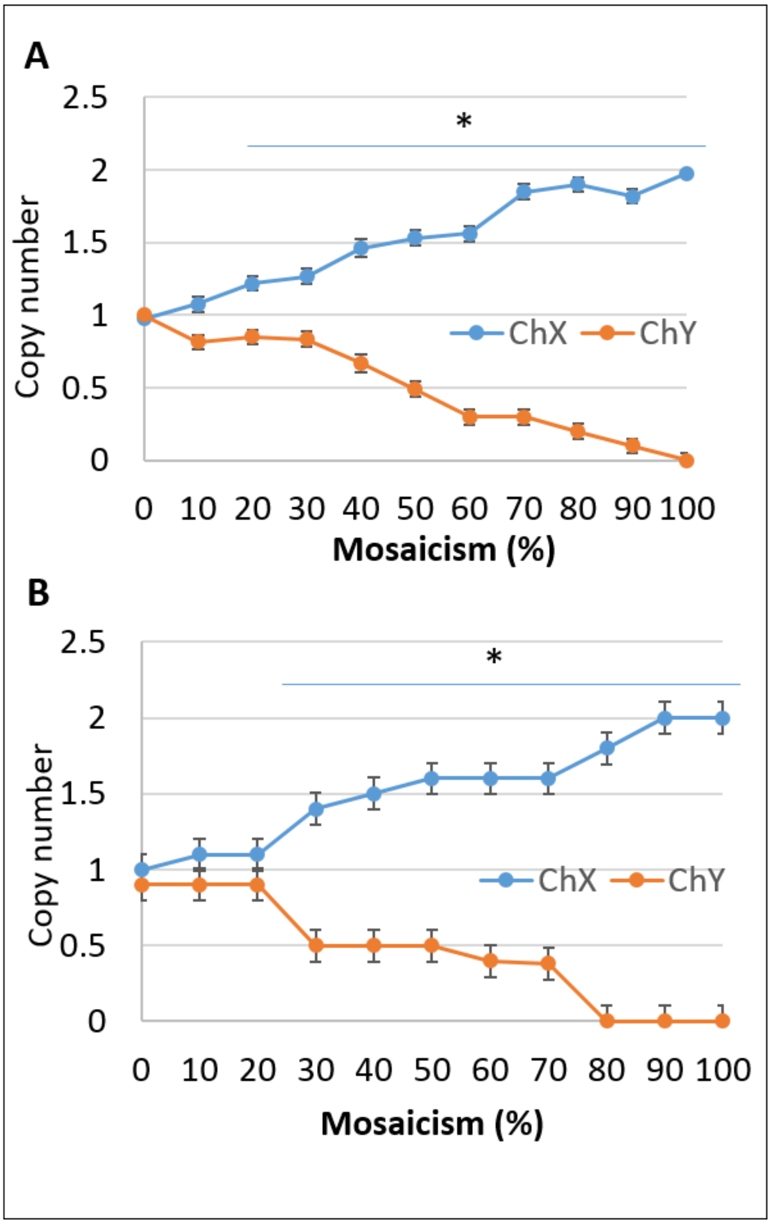

Figure S1. Reference curves obtained with reconstructed samples. The copy number of each dots (i.e., read count) for the mosaic chromosome was measured and the average value ± SD was correlated with the percentage of aneuploid cells present in each reconstructed samples. A: result from MiSeq (VeriSeq); B: results from Ion PGM (Reproseq). Reference curve obtained after the analysis of mosaic samples composed of a mix of cells with XX and XY karyotype. Orange line= ChY and blue line ChY. * $p < 0.05$ compared to euploid sample.
